# A Dishful of a Troubled Mind: Induced Pluripotent Stem Cells in Psychiatric Research

**DOI:** 10.1155/2016/7909176

**Published:** 2015-12-29

**Authors:** Sára Kálmán, Edit Hathy, János M. Réthelyi

**Affiliations:** ^1^Department of Psychiatry, Faculty of Medicine, University of Szeged, Szeged, Hungary; ^2^National Brain Research Program, Hungarian Academy of Sciences, Molecular Psychiatry and In Vitro Disease Modeling Research Group, Budapest, Hungary; ^3^Department of Psychiatry and Psychotherapy, Faculty of Medicine, Semmelweis University, Budapest, Hungary

## Abstract

Neuronal differentiation of induced pluripotent stem cells and direct reprogramming represent powerful methods for modeling the development of neurons *in vitro*. Moreover, this approach is also a means for comparing various cellular phenotypes between cell lines originating from healthy and diseased individuals or isogenic cell lines engineered to differ at only one or a few genomic loci. Despite methodological constraints and initial skepticism regarding this approach, the field is expanding at a fast pace. The improvements include the development of new differentiation protocols resulting in selected neuronal populations (e.g., dopaminergic, GABAergic, hippocampal, and cortical), the widespread use of genome editing methods, and single-cell techniques. A major challenge awaiting *in vitro* disease modeling is the integration of clinical data in the models, by selection of well characterized clinical populations. Ideally, these models will also demonstrate how different diagnostic categories share overlapping molecular disease mechanisms, but also have unique characteristics. In this review we evaluate studies with regard to the described developments, to demonstrate how differentiation of induced pluripotent stem cells and direct reprogramming can contribute to psychiatry.

## 1. Challenges in Psychiatric Research

Schizophrenia (SCZ) and bipolar disorder (BPD) present with overlapping clinical symptomatology and share many environmental and genetic risk factors (for a review see [1]). Additionally, extended literature argues on the neurodevelopmental origin and neuroprogressive course of both syndromes. Although psychotic disorders and BPD are not the most frequent psychiatric conditions, they affected 8 million people in Europe and costed €125 billion for the society in 2010 according to the report of the European Brain Council [2].

As mental disorders are exclusively human conditions, investigating and modeling these conditions raise several problems and necessitate compromises. Animal models, based on rare mutations of large effects, provide valuable information on the cellular biology and behavioral endophenotypes of psychiatric disorders but obviously have their limitations and validation difficulties. Indeed, 92% of drugs that passed preclinical studies fail in the clinical phase due to lack of efficacy or safety reasons [3].

*In vivo* brain sampling in psychiatric patients or control, healthy subjects is ethically and technically problematic. Postmortem tissue samples are widely used for assessment of architectural and molecular alterations in brain disorders, but the results must be evaluated circumspectly regarding the variability in the sampled brain area, the pre- and postmortem circumstances and the consequent degradation of RNAs and proteins. In order to countervail these technical issues, brain banks provide great sample sizes, standardized methodology, and detailed clinical information; however, these samples are not appropriate for functional assays or diagnostic purposes and the observed changes might be evoked by comorbidities or environmental factors over the course of the disease.

The heritability of SCZ, BPD, and autism spectrum disorders (ASD) is above 80% [4–6], but neither candidate gene nor genome-wide association (GWA) studies can fully explain this magnitude. These hidden genetics substantiated the theory of rare mutations with large effects versus common alleles with low penetrance [7]. Accordingly, in most of the cases psychiatric diseases are multifactorial and thus derive from the constellation of (otherwise harmless) common susceptibility alleles and environmental factors. Cases caused by single mutations occur very rarely and remain undetected in large-scale studies. Additionally, recent studies suggested that* de novo* mutations may have a great impact on the individual susceptibility [8, 9].* In vitro* cell culture models represent a system-oriented view, in which mental disorders are the manifestations of the donor's individual genetics, and along this line they enable performing functional assays to map gene × environment (G × E) and gene × gene (G × G) interactions.

## 2. Manufacturing Neurons: Made in Dish

**Figure 1:**
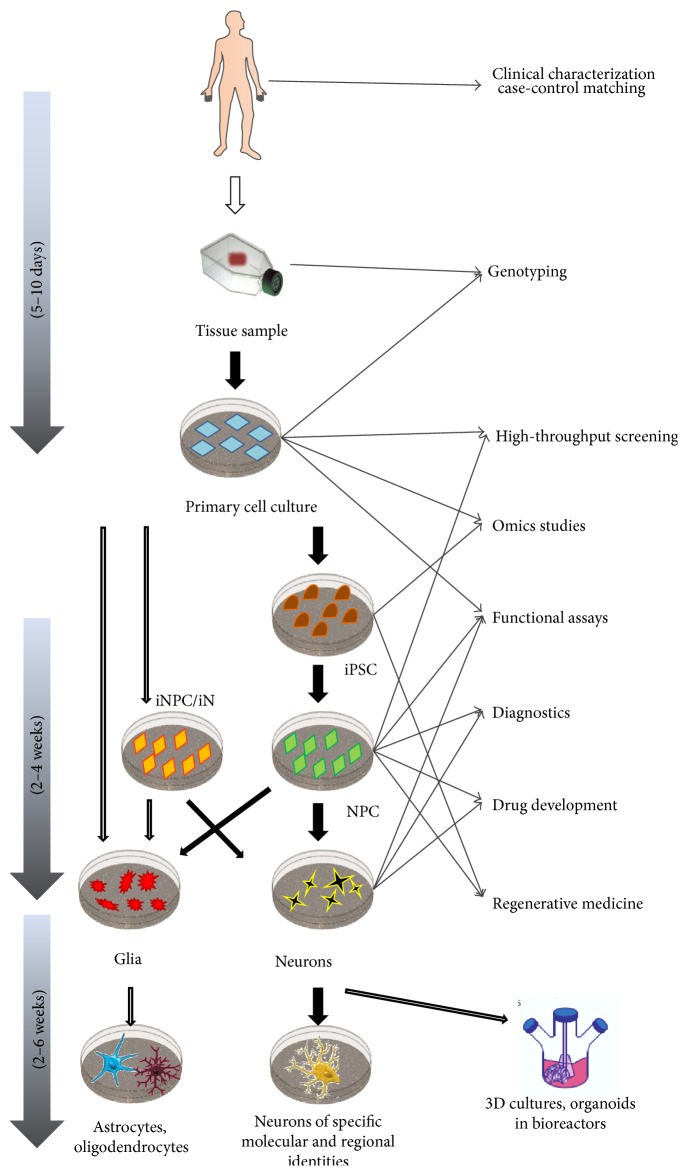
Schematic illustration of induced pluripotent stem cell and neural cell line generation and further clinical and research applications. (iPSC: induced pluripotent stem cell; iNPC: induced neural progenitor cell; iN: induced neuron).

Since detailed description of the iPSC/iNC induction and differentiation would extend the limitations of this paper and several publications have been already written on this rapidly developing field, here we will only briefly summarize the main technical issues (Figure 1). For further information and comparison of different protocols see [92–94].

Currently, there are three methods to generate human neural cells* in vitro*: iPSCs can be differentiated into neuronal progenitor cells (NPCs) and somatic cells can be transdifferentiated into neural stem cells or directly into neurons. Interestingly, transdifferentiation experiments have not yet been performed in the context of psychiatric disorders, even though four transcription factors or two microRNAs are enough to convert human fibroblasts into functional neurons within three weeks [95, 96]. Direct conversion has the advantage of bypassing the prolonged, potentially mutagenic phases of reprogramming and intensive progenitor proliferation [97]. On the other hand, the amount of experimental material is limited by the number of somatic cells and the efficacy of the transdifferentiation.

Somatic cells can be reprogrammed into pluripotent stage with a set of transcriptional factors, namely, OCT4, SOX2, KLF4, and c-MYC or OCT4, SOX2, NANOG, and LIN28 [98, 99]. These can be entered into the cells via integrating (lenti- and retroviral) or non-integrative (adenovirus, Sendai virus, episomal vector, and synthetic mRNA) vectors or direct protein delivering tools [100]. After initial induction, endogenous* NANOG*,* SOX2*, and* OCT3/4* expression indicate pluripotency which can be maintained via basic fibroblast growth factor (bFGF) supplementation for theoretically unlimited time.

The differentiation of iPSCs is thought to follow* in vivo* developmental pathways and require environmental cues. During the past eight years several protocols have been developed based on monolayer dual SMAD inhibition [101] or embryoid aggregates [102] with an efficacy of 80% or more than 85%, respectively. (For a comparative review see [103].) Successfully differentiated or transformed cells can be easily recognized by the detection of PAX6, an early forebrain neuronal marker. Since embryonic aggregate-based techniques reduce the variability of differentiation potential among pluripotent cells, it results in a more homogenous cell population. However, the culture always contains progenitors, glial cells, and mature or immature neurons with different neurotransmitter and receptor profiles and varying electrophysiological properties [13].

During manufacturing specific neurons, two major approaches are available. (1) High neurotransmitter specificity can be evoked by viral vectors or the combination of growth factors/small molecules. GABAergic cortical interneurons [104, 105], dopaminergic midbrain neurons [106, 107], dentate gyrus granule cells, and glutamatergic pyramidal neurons were successfully generated according to these protocols with an efficacy above 90%. (2) On the other hand, one can address the investigation of region and layer-specific neurons. Remarkably, NPCs emerging from neural rosettes show self-organized spatiotemporal differentiation pattern and model the six-layered cortical structure. Benefitting from this, researchers are able to cultivate and isolate early and late cortical progenitors, preplate neurons, deep (V-VI) and superficial (II–IV) layer neurons in a definite temporal manner [108]. Furthermore, advents of biotechnologies already allow us to grow neural and glial cells in 3D cultures, forming functional organoids which resemble certain brain regions on the cellular and tissue level [109]. These microphysiological systems could be especially valuable tools for modeling neurodevelopmental or neuroprogressive diseases.

The basic requirements against differentiation are as follows: quick and efficient generation of homogenous cell populations with physiological (or diseased) characteristics. The main concern about currently existing protocols is that they frequently result in heterogeneous, asynchronic cell populations with varying phenotypes and maturation states [13]. Remarkable efforts are made to develop cost-effective, large-scale generation of NPCs and matured, synchronized neurons for high-throughput assays [14, 110].

## 3. Closer to Perfection or Just Misperception?

iPSC/iNC cultures as model systems have advantages and disadvantages: (1) the cell lines can be initiated from easily obtainable biospecimens, for example, blood sample, skin biopsy, hair follicle, or urine, (2) but the reprogramming, culturing, and differentiation are labor intensive and require notable expertise [111]. (3) iPSCs and iNPCs have self-sustaining capacity, (4) while differentiated neurons are in postmitotic state; hence they provide restricted experimental material. (5) During reprogramming and differentiation the cells undergo epigenetic rearrangement, (6) and proliferating iPSCs exhibit genetic instability which may result in population diversity and biases in genetic assays [112]. (7) Neurotransmitter and brain region specific neurons provide unique opportunity to study the pathophysiology and genetics of neuropsychiatric disorders, (8) but every new protocol has to be carefully validated regarding the cell type-specific markers and the analogies and discrepancies compared to* in vivo* and animal model findings. (9) Finally, iPSC/iNC experiments are time and money consuming.

## 4. Three Years' Balance: Debits and Credits

In 2012, the National Institute of Mental Health (NIMH) and the Foundation for the NIH organized a workshop on the technological advances and challenges of patient-derived iPSCs/iNCs research in psychiatry. Scientists and delegates of industry and government and funding organizations conceived recommendations and directions for the future focusing on basic research, new target identification, and drug development (for the meeting report see [10]). Since stem cell biology and brain research are two of the most active fields of life sciences, we decided to review systematically the literature on iPSC/iNC in the context of psychiatric diseases and strike a balance: Did we take our own advice? How far have we got? What challenges do we face? (Table 1).

To give exact answers, we performed, first in the literature, a systematic review on iPSC/iNC based researches in psychiatry. We searched PubMed with the following keywords: “induced pluripotent OR reprogramming OR transdifferentiation AND psychiatry OR schizophrenia OR bipolar OR major depression OR autism” until 20 June, 2015. First, we catalogued research papers, if (at least part of) the experiment was conducted on human induced pluripotent derived or transdifferentiated neural cell cultures and English text was available. We excluded papers on neurodegenerative disorders, dementias, and epilepsies (Table 2(a)). During the survey, we noticed that review publications display outstanding proportion of the literature (41%); thus we decided to list the relevant reviews using the same criteria (Table 2(b)).

## 5. What Can (Should) Be Reviewed?

**Figure 2:**
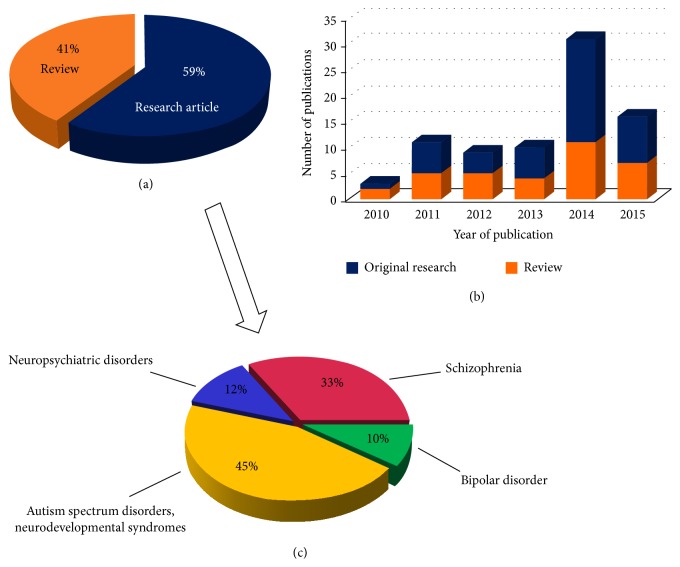
(a), (b), and (c) Characteristics of current literature dealing with induced pluripotent/neural cell lines in psychiatric research. (c) Represents the research articles only. Publications were reviewed until June, 2015.

After categorizing the items, we found 80 relevant publications: 48 research articles and 33 reviews (Figures 2(a), 2(b), and 2(c)). Intriguingly, we did not find any papers with iPSCs/iNCs targeting major depression. This is surprising, since unipolar depression is the leading chronic disorder in the WHO European Region, and the third cause of disability-adjusted life-years (DALY) [113], while only half of the patients receive adequate therapy with 70% long-term efficacy [114]. Considering that major depression is a multifactorial, neurobiological disorder with heritability estimates between 40 and 60% [115] and peripheral cells were traditionally used to model and study the diseased endophenotypes [116–118], we can envisage that iPSCs/iNCs hold great promise in this field.

## 6. Evidence-Based Questions

iPSC/iNC studies are interpretable only in a multiscale disease modeling paradigm; thus results from a well-designed experiment should raise more questions than those they answer* per se*. Therefore, we examine the main issues addressed at the 2012 NIMH workshop [10] and those which emerged since* via* introducing some of the published studies and focusing on answered and emerged future challenges.

### 6.1. Time Is Not Everything, but Timing!

The 2012 workshop highly emphasized the need for effective, standardized derivation and validation protocols for high-scale studies and future clinical application of iPSC/iNC technologies [10]. The participants cautioned against proliferation of poorly designed studies and recommended the establishment of rigorous, transparent, and reproducible methodology.

Reviewing the literature, we can conclude that significant progression was made in neural differentiation methods during the past three years. Today, researchers are able to generate neurons of regional and temporal identity, as well as 3D organoids; however, the methods are still under intense development for more efficient, time-sparing protocols. Since we have to allow room for these innovations, validation practices call for standardization.

Key questions are as follows: What are we modeling? (Which developmental and functional state of the* in vivo* neurons?) What kind of indicators and assays should be used for quality control? iPSCs are considered almost undistinguishable compared to human embryonic stem cells; and iPSC derived NPCs form rosettes, analog to the neural tube, the embryonic primordium of the central nervous system [119]. Thus, it is reasonable to assume that we are modeling fetal neurogenesis and neurodevelopment in cell cultures under differentiation. Accordingly, Brennand et al. found that the gene expression profile of NPCs and even 6-week-old differentiated neurons resembles the first-trimester fetal brain at the most [16]. Moreover, the electrophysiological properties of stem cell derived neurons share common temporal pattern with pyramidal neurons of the postnatal animal neocortex [120].

However, we have to declare that our knowledge on pre- and postnatal brain development is restricted and mainly relies on animal and human fetal brain studies. This limited insight and the* in vitro* observations underpin that iPSC differentiation and iNC maturing follow the* in vivo* timeline and stages and react to the same exogenous effects [93].

For instance, Boissart et al. demonstrated that the differentiational potential of iNPCs follows a temporal manner similar to what has been previously described in animal models [14]. The group delayed cellular commitment with high-mitogenic medium and sustained NPC proliferation for passages 8–20. The prolonged proliferation period resulted in a homogeneous late cortical progenitor population that spontaneously differentiated into superficial cortical neurons. Three weeks after withdrawal of mitogenic factors, more than 80% of the cells were glutamatergic. Importantly, the short, synchronous, and highly productive differentiation period makes this method amenable to high-throughput assays.

### 6.2. Doing Well in the Wells

Neurons, specified from progenitor cells, are immature and require 4–12 weeks to reach their definitive phenotype* in vitro*, which [119, 121, 122] is a notable hampering factor of iNC studies. Literature proves that detailed investigation of this period is crucial for the following technical and validation reasons.

(1) First, transition from pluripotency to differentiated neuronal state is attended by complex, pervasive gene expression changes. Fathi et al. analyzed the differentiation-related transcriptome dynamics and revealed that 5955 transcripts were modified during the 4-week-long protocol [123]. Of note, 2589 transcripts were upregulated in the differentiated neurons compared to pluripotent cells. On one hand, this intensive, early period can be used to unveil and understand neurodevelopmental disturbances and bridge between disease-associated genotypes and endophenotypes. On the other hand, experimental design, timing, and theories must be set up circumspectly.

(2) Differentiation and maturation can be influenced by the culturing conditions; therefore, several researches proposed protocol modifications to shorten the “before-the-experiment” period and improve synchronicity. For instance, Tang et al. compared the neural maturation on two different surfaces: the most frequently used artificial coating, laminin, versus astrocyte layer [124]. Astrocytes promoted the differentiation, soma and neurite growth, and dendrite arborization. They supported functional maturation with regard to ion channel and receptor expression and synaptic transmission. Still, even if cultured on astrocytes, neurons did not exhibit matured synaptic activity before the third week, and cells on laminin were further delayed. Astrocyte-conditioned [125], ascorbic acid [126] and cAMP supplemented [123] culturing medium or the application of graphene oxide nanomaterial [127] also accelerates differentiation.

(3) We also cannot pass by the fact that the extended, responsive maturation may lead to phenotypic heterogeneity within the dish and result in a mix of progenitors and immature, partially or fully matured neurons in different ratios [13]. Additionally, NPCs and neurons display similar appearance and NPCs express glutamate [128], GABA [129], and dopamine [130] receptors.

### 6.3. Casting Neurons

Given the above-mentioned issues, we suggest that every iPSC/iNC experiment should include the developmental and functional characterization of the subject cells. The main approaches for describing a neuron are as follows: neural marker detection, receptor and ion channel profiling, electrophysiological analysis, and the evaluation of synaptic functions via enzyme activity, neurotransmitter release, metabolism, and reuptake [131].

The methodology of neural marker detection developed concomitantly with the differentiation protocols. Today, we are able to generate and identify cortical excitatory glutamatergic pyramidal cells [102]; GABAergic inhibitory interneurons [105]; cerebellar Purkinje cells [122]; or dopaminergic neurons of the substantia nigra [131]. However, the presence of the differentiation markers is not indicative of the neuron's maturation state.

The work of Dage et al. revealed that detailed pharmacological characterization of the differentiated neurons would be much desired since the receptor and ion channel signature of differentiated neurons may change almost day by day [24]. Furthermore, maturing iPSC-derived neurons do not display NMDA receptor subunit switch, peculiar to the neonatal brain [24], which might be a relevant difference between* in vitro* and* in vivo* fashions.

Data suggest that electrophysiological assessment of the cells might be the most potent approach for defining maturation states. The electrophysiological development* in vitro* resembles postnatal neocortical changes observed in animal models. Namely, the resting membrane potential becomes more negative, the duration of action potential decreases, and the Na^+^, K^+^, and Ca^2+^ currents show time-dependent changes [120]. The milestones are thought to be the appearance of spontaneous excitatory postsynaptic currents (sEPSC) and action potentials in fresh neurons [122] and capability for repetitive action potentials in matured neurons [13, 120, 131].

Belinsky et al. went further. They carried out patch clamp, Ca^2+^ imaging, and PCR on single-cell level to correlate the electrophysiological properties and the gene expression pattern of differentiating and maturing neurons [13]. The cells demonstrated action potential already from day 15, but expression of several neuronal physiology and disease-associated genes were delayed until day 29 (*COMT*,* DISC1*,* DTNBP1*,* GAD1*, and* PAX6*). These thought-provoking results press for multimethod validation. Recently, Chatzidaki et al. [132] demonstrated the detailed pharmacological characterization of nicotinic acetylcholine receptors on iPSC-derived human neurons. The exact time points of the functional and developmental stages highly depend on the cell type and the culturing protocol; therefore, an orientating timeline with the defined, critical landmarks and minimally required validation assays could assist the interpretation and reproducibility of* in vitro *findings. Furtherly, high-throughput assays offer the possibility to monitor the cell cultures around the clock, analyze structural and functional alterations (e.g., synaptic activity), and capture the right time to run hundreds of tests.

### 6.4. Neurons, into Single File!

We are only beginning to realize the magnitude of heterogeneity in neuronal cultures derived from iPSCs. Earlier lines of evidence already highlighted the fact that, despite the utilization of targeted differentiation protocols, neuronal cultures remain heterogeneous and give rise to mixed populations of glutamatergic, GABAergic, and dopaminergic neurons, as well as astrocytes and undifferentiated cells. Recently, the development of single-cell approaches makes it possible to determine the ratio of neurons versus astroglia, neuronal subpopulations, or neurons reaching a specific stage of maturation, within a population of differentiating neurons. Neuron-to-neuron variation seems to be a general feature of* in vitro* as well as* in vivo* neuronal populations. Moreover the degree of variation also casts light on faulty neurodevelopment associated with neuropsychiatric disorders. Shcheglovitov et al. [48] used single-cell methodology to analyze neuronal cultures derived from patients suffering from Phelan-McDermid syndrome, a rare condition caused by 22q13.3 deletion. Besides other major findings, this outstanding paper also points out that only a small proportion of neurons express postsynaptic density proteins SHANK1–SHANK3, indicating small fraction of synaptically mature neurons. 20–60% of neurons expressed TBR1, CTIP2, and SATB2 upper layer cortical markers, while less than 10% of neurons expressed GABAergic markers. In their methodological summary, Citri et al. [133] provide a protocol for single-iN qPCR and present data indicating the low proportion of VGLUT1 and VGLUT2 expressing iNs, suggesting delayed synaptic maturation in this system [134]. The described single-cell methodology has been successfully incorporated in several other studies. While neuronal differentiation protocols are much better characterized in recent years, the above findings illustrate that single-cell approaches are on the rise and will be important and necessary tools in the armamentarium of* in vitro* neuronal disease modeling efforts.

### 6.5. Thinking Big

Theoretical concepts of psychiatric disorders changed radically in the recent decades: the immune-neurodevelopmental model of SCZ and ASD [135], neuroinflammation-degeneration theory of MDD [136], and the need for G × E and network-based diagnostic and therapeutic approaches became widely accepted. But the revolution is still delayed in psychopharmacology. One detrimental factor can be the lengthy, expensive, and animal model based testing of potential new targets and compounds. Therefore, robotic high-throughput assays on human cell lines could be milestones in the paradigm shift towards human biology based,* in vitro* drug development [137]. iPSC/iNCs can be optimal subjects for these studies.

Fragile X syndrome is neurodevelopmental disorder caused by a silencing mutation of the* FMR1* gene. Recently, two independent research groups developed and published high-throughput screening methods for FMR protein detection and novel drug identification using patient-derived iNPCs [33, 138]. Kumari et al. designed a time-resolved fluorescence energy transfer (TR-FRET) based assay to measure FMRP levels in 1 536 wells and screen 1 280 pharmacologically active compounds parallel to identify those which increase the expression of the silenced gene [33]. The most effective molecules were retested in a secondary assay using qRT-PCR and further confirmed by dose-response experiment. Kaufmann et al. used high-content screening to observe cell morphology and FMRP expression alterations and tested the efficacy and toxicity of 50 000 compounds [138]. Importantly, automated high resolution microscopy and machine learning algorithms allow single-cell-based follow-up and data analysis in living cultures; therefore the group was able to detect that a remarkable subpopulation of the cells (40%) responds to certain drugs, although the averaged measures did not reach significance.

Both works demonstrated a sensitive, cost-effective approach for drug development. However, we have to add that the utility of progenitor cells is frequently suboptimal or invalid in psychopharmacology; and current high-throughput methods are optimized for proliferating, easily transferable cell lines and not for postmitotic neurons [42, 139]. To reduce the technical difficulties, Niedringhaus et al. worked out a transferable raft miniculturing practice for neuron cultures which provide sufficient experimental material for microvolume reactions and appropriate sample sizes [42]. Notably, the cell viability, sample-to-sample reproducibility, and screening potential proved to be higher than those on conventional well-plates.

An additional hampering factor might be the time-consuming derivation and characterization of neurons from somatic cells accompanied by several technical pitfalls. Research industry offers several genetically modified iPSC-derived neurons which can be a faster model for drug development after thorough validation [125].

### 6.6. Double Standard: Stable and Flexible

The iPSC/iNC line generation requires stem cell-like, incompact chromatin and multiple epigenetic rearrangements. Controversially, scientists agree that a high-quality iPSC/iNC assay conserves the donor's genetic information (including parental imprinting). The NIMH meeting participants, and many since then, conceived a reasonable concern on the genetic (un)stability of induced cell lines [10].

Previously, chromosome aberrations, gene deletions/duplications, and point mutations were thought to be the main mechanisms responsible for* de novo* mutations during the pluripotent state. However, multiple studies demonstrated that iPSCs and iNPCs exhibit chromosomal stability (one examined 1700 stem cell cultures [140]). Per contra, copy number variations (CNVs) show significant incidence [23, 112]. All of the investigated cell lines gained CNVs and the CNV signature of the iPSCs differed from both somatic parent cells and iNCs. This indicates that* de novo* mutations may appear through the somatic cell-iPSC-NPC-neuron transitions. On the other hand, mosaicism for CNVs is also common* in vivo*: it represents 0.12–7.3% of intraindividual genomic variability [141].

Keeping in mind that CNVs are known to influence clonal selection in cell cultures [142] and participate in the etiology of several human diseases, including neurodevelopmental disorders (SCZ [143], ASD [31], and ADHD [144]), the iPSC/iNC quality control calls for reconsideration. It was always supposed but, recently, Kang et al. proved that genetic integrity is highly influenced by the reprogramming technique, and DNA nonintegrating protocols are safer [145]. The previously recommended karyotyping is not sufficient for ensuring validity; instead, DNA sequencing of the source and generated cells is desirable.

### 6.7. The Neuronal Genome: Imperfectly Imitable?

Somatic mosaicism affects the brain also: brain-only or brain region-only somatic mutations, chromosome aneuploidy, microdeletions, or retrotransposon dynamics have been detected in post mortem brain tissue. Presumably, these neural genomic variations contribute to the functional heterogeneity of brain cells and also to the development of neuropsychiatric disorders [146]. For instance, L1 retroelements (the only human retrotransposon with autonomous activity) exhibit increased copy number in adult NPCs compared to nonbrain cells and are known to be associated with Rett syndrome [147]. Bundo et al. examined the L1 signature in SCZ using multiple model systems [18]. First, they showed that L1 copy number is increased in the prefrontal cortical neurons of SCZ patients compared to controls and autologous nonbrain cells. To answer the question whether this ensues from hereditary or environmental factors, the group assessed the L1 profile in an environment-induced SCZ animal model and in iNCs derived from SCZ patients with rare mutation of large effect (22q11 deletion). The genomic DNA of the mouse brain exhibited the consequences of high L1 activity and they also detected increased L1 insertion rate during* in vitro* neurogenesis. Moreover, brain-specific L1 insertion sites were near or in genes involved in synaptic functions and neuropsychiatric disorders supporting the possible pathognomic role of retrotransposition events. For conclusion, we can speculate that increased L1 dynamics by environmental and/or genetic factors may increase the susceptibility to neurodevelopmental disorders by disrupting synaptic and schizophrenia related genes in neurons.

This outstanding experimental design reassures the validity of iPSCs/iNCs in modeling neurodevelopment and calls for further studies on the neuronal genome.

### 6.8. Designation of Origin

Besides the above-mentioned somatic mosaicism, cellular commitment and* in vivo* cell-aging raise the following question: Does the source cell type matter? Neuropsychiatric studies apply the most frequently easy-to-obtain lymphocytes, fibroblasts, and keratinocytes; but, theoretically, all somatic cells can be reprogrammed or transdifferentiated into iPSCs and iNCs.

These somatic cells widely vary in their epigenetics, proliferative potential, and the rate of cellular aging. Previous studies found that the intrinsic properties of the source cells, that is, stage of differentiation [148], senescence [149], tissue type [150], and number of passages [151], influence the efficacy of reprogramming. Furthermore, the generated cells display a residual gene expression pattern of the source cell type referring to “incomplete reprogramming” and result in variability among iPSCs from different tissue samples [152].

Chen et al. speculated that induced cell lines may retain and transmit transcriptional/epigenetic marks from the source tissue to the differentiated cells; therefore, they carried out whole transcriptome analysis in neurons gained from fibroblasts or dental pulp [20]. Notably, they found 63 differentially expressed genes, including a glutamate receptor, choline, GABA, and glycine transporter. Striking differences were found in the expression of the* SLITRK2* gene (associated with BPD and ASD), multiple* HOX* genes, and a set of transcription factors. Pathway analysis revealed that neurological disease/schizophrenia related gene sets are the most affected [20]. Unfortunately, the interpretation of the results might be problematic since the dental pulp was obtained from a 12-year-old male subject, while fibroblasts originated from a 30-year-old female and a 58-year-old male. However, the experiment is highly noteworthy: even if we conclude that the source cell type or the donor's age passed down through generations and affected the gene expression of the iNCs, it urges for further research.

### 6.9. Patients Ill Sorted?

One of the main research principles is representativeness: the sample has to be an unbiased illustration of the studied population. Psychiatric diagnostic categories cover a wide range of patients with great heterogeneity in etiological factors, symptomatology, disease progression, and therapy response. This diversity issued several difficulties in experimental and clinical settings during the past decades.

Now, the overlook is changing: DSM-5 omitted the previous SCZ subtypes defined by clinical symptoms [153]. Two years later, the Consortium on the Genetics of Schizophrenia (COGS) postulated that endophenotypes could provide a more negotiable approach of patient categorization for clinical and research purposes [154]. Endophenotypes, for example, cognitive dysfunction, EEG-markers, or brain imaging phenotypes, are quantitative laboratory based measures with a same level of heritability as SCZ itself [155]. They can be linked to certain genotypes, cellular phenotypes, psychopathologies, and functional impairments and fill the gene to phene gap. The NIMH workshop in 2012 also addressed the critical step of patient selection and recommended subject recruitment based on comprehensive clinical, genetic, and cellular characterization.

### 6.10. The Art of Design

Disrupted in schizophrenia 1 (*DISC1*) gene is known to be involved in fetal and adult neurodevelopment and neuroplasticity, and its variations are highly associated with a wide range of mental disorders: SCZ, BPD, MDD, and ASD as well [156]. Presumably, it predisposes to endophenotypes which can manifest themselves in different clinical syndromes depending on the genetic and environmental cofactors [157].

Wen et al. aimed to study cellular consequences of the DISC1 mutation [54]. They derived iPSC lines from a carrier pedigree: one SCZ and one MDD patient with a DISC1 frameshift mutation, two unaffected family members without the mutation, and one additional control: an unrelated healthy subject. The DISC1 mutation caused functional synaptic transmission deficits in the glutamatergic neurons* via* pervading transcriptome alterations. To challenge their hypothesis on the primary pathognomic effect of the DISC1 mutation, the research group repeated the measures with the isogenic pairs of the cell lines, generated by DNA editing. Over and above the obvious values of their results in understanding DISC1 related pathophysiology and filling the gap between the genotype and the clinical picture with a cellular endophenotype, this work provides a great example for careful, overthought study design.

Considering that patient iPSC/iNC studies are usually conducted on small sample sizes (1–5 persons/group) and advancements are trending towards personalized medicine, careful case-control matching in iPSC/iNC research is crucial to maintain validity and reliability. When monogenic diseases (e.g., Rett and Fragile X syndrome) are investigated, the gender, age, and/or genotype based selection of healthy individuals is sufficient. Per contra, in case of polygenic neuropsychiatric disorders with intermediate heritability (30–70%) other designs are noteworthy since the combined effect of all disease-related and irrelevant alleles manifests itself in the dish [158].

For instance, pedigree-studies allow identifying heritability factors. Inclusion of affected and unaffected family members reduces the genetic noise (heterogeneity) and thus the risk of type I error. While a second, independent control group from out-of-pedigree healthy subjects helps to oversee the potential effects from family genetic background. Isogenic pairs produced by genetic correcting technologies (e.g., TALE nuclease [159], zinc finger nuclease [160], homologous recombination [161], or CRISPR [162]) allow a cell line to be its own control and enables the targeted testing of single gene effects via excluding (epi)genetic diversity. In some cases (e.g., CNVs, trinucleotide repeats, and X-linked disorders),* in vivo* or* in vitro* mosaicism provides isogenic controls [26].

### 6.11. Psyche in the Dish?

The question is not sceptic by all means. How can we validate a specific iPSC/iNC line for modeling a psychiatric disorder? Chromosomal and cellular marker characterization and evaluation of genetic stability are only the base of quality control. Having differentiated neurons does not evidence a model for neurophysiology or disease neuropathology.

Two research groups aimed the validation of a commercially available neural cell line for further studies on ASD and neurodegenerative diseases [1, 24, 125]. The corroboration included whole transcriptome analysis, receptor and ion channel profiling, and detailed electrophysiological characterization. According to their results, the cells display early developmental neural phenotype and express the majority of ASD associated genes and thus this cell line might be utilized as control in comparison to patient-derived cells or suitable for isogenic mutant—wild type pair generation.

Interestingly, many of the ASD-related genes showed changes along the culturing process [24], and Belinsky et al. also detected specific temporal manner in the genes of interest (DISC1, DTNBP1, GAD1, PAX6, FOXP1, FOXP2, vGLUT1, and COMT) [13]. This sounds reasonable since the mentioned genes are linked to neurodevelopment, synaptic transmission, and intracellular signaling, functions highly implicated during neural maturing. However, these observations are worthy of note, suggesting that timing can easily enhance or undermine the validity of the experiment, especially when the most frequently used early neural and forebrain markers (PAX6 and vGLUT1 and GAD1) or canonical disease genes (DISC1 and COMT) are the subjects. Concordantly, Wen et al. found that DISC1 mutation caused structural anomalies and synaptic dysfunction attenuate around postdifferentiation weeks 4–6 [54].

Usually, experimental data can be embedded into previous results and legitimized by the cited literature. Considering the novelty of the field and the limited number of research papers, iPSC/iNC researchers can rarely expect their model's validation from preceding experiments. Comparing the results with animal model and human postmortem findings can offer pivots; however, dissimilarities might ensue from differences between species and cell types since we are matching pure neuron cultures with tissues [41].

### 6.12. Grafting Neurons for Psychiatric Treatment?

Three future applications of iPSCs and iNCs are most commonly predicted [24]. As model systems, they can help us understand the cellular pathophysiology of neuropsychiatric disorders and reveal genotype-phenotype correlations. High-throughput cellular screening assays may provide novel targets in drug development; and, finally, iPSC-derived cells may play a role in regenerative medicine.

Induced or embryonic PSC-based replacement therapy research is one of the most expeditiously developing fields of medicine. Currently, clinical trials are in progress in macula degeneration, type I diabetes mellitus, ischemic heart failure, and spinal cord injury [163]. Preclinical results are also promising in Parkinson's and Alzheimer's disease, neurodegenerative disorders with well-defined pathological and functional alterations and cellular loss (recently reviewed in [163, 164]). And what about other psychiatric disorders? Animal models can provide us with hints about this application.

For instance, SCZ has multifactorial origin which pervades the whole connectivity during neurodevelopment and results in poorly understood brain pathology. Human and animal research suggest that cortical and hippocampal inhibitory interneuron deficit contributes to the dopaminergic system dysfunction, thus the positive symptoms of SCZ [165]. In rodents, embryonic and induced human and nonhuman NPC grafts survive, proliferate, migrate, and differentiate spontaneously into pyramidal or GABAergic neurons [166–168]. The integration of these inhibitory neurons into the host neural circuits successfully modulated the hyperactive dopaminergic system and the behavior analogous of positive symptoms in a mouse SCZ model. While these engraftment experiments are perplexing and can contribute substantially to our understanding of the neurobiology of psychiatric disorders, they do not necessarily forecast engraftment of* in vitro* differentiated neurons as a feasible approach for the treatment of psychiatric disorders in the near future. Disease pathology remains poorly understood and the clinical hurdles are also numerous.

### 6.13. Open Access in Stem Cell Based Disease Modeling

The NIMH workshop addressed one more intensively discussed, still actual issue: information and resource sharing which is especially meaningful in the rapidly developing scientific fields such as iPSC/iNC research [10]. The participants argued that open sharing of data and standardization of iPSC/iNC generation and validation protocols are essential for improving experimental reproducibility.

It is well known that open sharing and collaborative environment empower knowledge circulation and thus fuels innovation and discovery. Therefore, decision-makers took significant steps to implement the open access policy: in 2015, both the European Commission and the US Congress supported the proposal that articles on publicly funded researches have to be freely available for anyone [169, 170]. We were curious if this open access movement is observable in the iPSC/iNC literature. We found that 73% of the reviewed research papers are freely accessible (35 from 48 articles, listed in Table 2(a)).

The other basis of open science is resource sharing. Growing number of cell banks collect somatic cells and/or iPSCs from patients with neuropsychiatric disorders and assure accessibility for the scientific community on request [17, 171]. Such (inter)national consortiums and collaborations provide standardized databases and methodology, increased, homogenous sample quality, and possibility to study rare genetic or phenotypic variations, affected families, or—as seen in Sweden—isogenic cell line pairs from monozygotic twins [172].

## 7. Tools in Our Hands: We Have Nothing to Fear

The ideal cell culture model for* in vitro* experiments meets the following requirements: (1) it is easily obtainable via minimal invasive intervention; (2) the initiation and maintenance of the cell culture are not extremely labor intensive or time-consuming; (3) the fact that differentiation, if necessary, can be directed and monitored; (4) The cell line reserves proliferative, self-sustaining capacity over passages; (5) and this is done with genetic stability; (6) it exhibits similar (or identical) pathophysiological features to the diseased tissue* in vivo*; and (7), last but not least, the method provides enough experimental material at reasonable expenses.

Revising the currently available* in vitro* systems, the words of Salvador Dali flash on: “Have no fear of perfection – you'll never reach it.” Ideal* in vitro* model cannot exist, but depending on the concept of the research and the accessible resources one can choose the most optimal from the following.

Extended literature discusses peripheral cells, such as blood leukocytes and dermal fibroblasts, as potential* in vitro* models and biomarker sources of mental disorders. They are relatively easy-to-obtain, robust cell lines and share receptor and signaling pathway similarities with CNS cells [173–175]. Fibroblasts have self-maintaining capacity* ab ovo* and maintain homogeneity between passages 5 and 20 [176]. Freshly isolated leukocytes are appropriate even for bedside functional assays or can be immortalized for culturing but represent poor genetic stability [177]. Finally, we cannot disregard that peripheral cells do not permit the examination of specific neural phenomena (e.g., electrophysiology, microarchitecture, and neurodevelopment).

Human primary neural cultures, initiated from brain biopsies, are barely applied to neuroscience. They require special conditions and lack self-maintaining capacity; therefore, the amount of experimental material is restricted. Furtherly, the cells are already tainted with life-long* in vivo* effects, which balks the expression of the naïve endophenotype.

Since 2006, when Takahashi and Yamanaka showed that the expression of four transcription factors can reprogram adult somatic cells into an earlier ontogenic state [98], induced pluripotent stem cells (iPSCs) and iPSC-derived cell lines became one of the most studied and advancing fields of medicine; but, as seen above, we still face numerous technical or theoretical issues.

## 8. Can We Make a Long Story Short?

The main concerns about iPSC-research, that is, time and resource demands, genetic instability, epigenetic changes, and populational heterogeneity, might be partially overcome by omission of the pluripotent state and direct transdifferentiation of somatic cells into completely different cell types. After Takahashi and Yamanaka introduced the method of cellular reprogramming, researchers started to work on new protocols to establish neural cultures from somatic cells by direct cell lineage conversion. Vierbuchen et al. were the first who successfully transdifferentiated mouse fibroblasts into functional neurons [95]. Since then, they and others showed that human somatic cells (e.g., fibroblasts [134], blood cells [178], and hepatocytes [179]) can be transdifferentiated into neural progenitor cells [180] or postmitotic neurons via forced expression or exogenous addition of transcription factors, microRNAs, or small molecules. Moreover, one single factor is enough to induce neural cell fate [181] and cell type-specific factors allow the generation of dopaminergic [182], motor neurons [183] or oligodendrocytes [184]. The induced neural cells resemble* in vivo* neurons in their functional, electrophysiological, translational characteristics and form functional synapses; thus, they can be valuable* in vitro* models of neuropsychiatric disorders. However, as of yet only one experiment has been published on direct reprogramming in the context of psychiatric disorders. Wang et al. [53] used lentiviral transduction with a construct expressing miR-9/9^*∗*^-124,* NEUROD2*,* ASCL1,* and* MYT1L *to transdifferentiate fibroblasts from bipolar patients responsive or unresponsive to lithium medication, the gold standard of mood-stabilizing treatments. iNCs derived from lithium-responders and lithium-nonresponders demonstrated different cell adhesion characteristics. This innovative approach demonstrates the translation potential and “nearly bedside” application of transdifferentiation-based assays.

The greatest advantage of direct lineage conversion is bypassing pluripotent states and avoiding potential oncogenicity as a consequence. On the other hand, this stability, that is, lack of self-renewal and potential amplification, might be a drawback in laboratory research and regenerative medicine. Besides, the possibility of residuals from* in vivo* cellular senescence and epigenetic memories inherited from the parental tissue deserves further considerations [185].

## 9. The Undeservedly Neglected: Glia-Associated Pathologies

Vast majority of psychiatric research deals with neurons but they account for only about one-third of human brain cells. Glial cells are responsible for maintaining brain homeostasis and neural well-being. They provide lactate as source of energy, regulate the redox balance, metabolic clearance, and CNS immunology [186, 187], and play crucial role in directing neuronal migration, neurite outgrowth, and synaptic pruning [188]. Additionally, astrocytes are in bidirectional cross talk with neurons, and glial neurotransmission has been proposed to be involved in several mental functions (e.g., memory, motor control, and decision making) and neuropsychiatric disorders [189]. Postmortem human and animal studies proved that glial cells contribute to the development and progression of psychiatric disorders and can be potential therapeutic targets [190, 191].

NPCs derived from iPSCs can be differentiated into glial precursors and mature astrocytes and oligodendrocytes [192, 193]. Microglial-like cells are even more easily obtainable due to their mesodermal origin, and circulating monocytes can be transdifferentiated within 14 days [194]. These cell lines are underrepresented in psychiatric research, only used as a feeder/supporting layer for the neurons. Per contra, they are intensively studied as potential disease models and therapeutical targets in neurological disorders, for example, Huntington disease [195], amyotrophic lateral sclerosis [196] or congenital hypomyelination [197], and intellectual disability [198].

Yet, we can find a great example: how neuron-glia cocultures can help to answer whether certain glia-associated pathologies are pathognomic factors, additive part of the endophenotype, or reactive (beneficial) response to the neural dysfunction. Williams et al. differentiated astrocytes and neurons from iPSCs of Rett syndrome patients and demonstrated that mutant astrocytes have non-cell-autonomous adverse effects both on cocultured mutant and on healthy neurons [55]. The group also proved that this impact is directed by the extracellular environment since the astroglia-conditioned media took the same detrimental effect on the morphology and function of wild type and mutant neuronal cultures. The adverse effects could be rescued by IGF-1 supplementation which underpins the ongoing IGF-1-based clinical trials in the treatment of Rett syndrome. Surprisingly, the efficiency of IGF-1 supplementation depended on the genotype of the astrocyte, which calls for further pharmacogenetic studies. According to these findings, the effects of the mutations in the astrocytes and neurons appear to be additive. Similar innovative studies will unravel the complex association of human neurons and glial cells in healthy and diseased brains.

## 10. Limitations

On the whole, results are reassuring. Those who reported differentiation and maturation anomalies in iNCs from patients with neurodevelopmental disorders [37, 41, 43, 45, 46, 49] are more than those who could not detect alterations during differentiation [14] or in electrophysiological properties [13, 54]. However, there are some noteworthy study design and publication biases.

(1) Scalability represents a central issue for stem cell based disease modeling. Ideally, the number of cell lines derived from specific patients could be scaled up to numbers typical for clinical studies, meaning tens or hundreds of patients. Realistically seen, today this is not feasible. However we must keep in mind that this method is in its infancy and the task remains the exploration of cell lines derived from genetically and clinically well characterized individuals.

(2)* A priori* hypotheses influence the objectives and the measured parameters. Differentiation is intensively monitored in these diseases; therefore, such small-scale variations like 12% nuclear size differences can be detected [11], but less is known about the transcriptome or receptor profile alterations. (3) Majority of the studies examined the iPSC/iNC lines at rest in monotonous environments which might be detrimental during neurodevelopment, that is, specialization for signal detection and transmission. Therefore environmental challenges could enhance and reveal additional differences as showed in [29, 36]. (4) Great proportion of these differences might affect synaptic transmission which is understudied in iPSC/iNC models; however, novel visualization and high-content screening technologies might bring advances to this field. For instance, functional assays of DISC1 mutant cells, namely, spontaneous synaptic current measurement and synaptic vesicle staining in living cells, revealed synaptic vesicle release defect which were also observable in the transcriptome [54].

(5) Similarly, there is a rate shift towards pervasive, monogenic neurodevelopmental syndromes with early-childhood presentation and well-defined clinical and genetical picture. The nature of these syndromes differs substantially from the most frequent adult psychiatric disorders which are multifactorial with less robust pathology and might show themselves in the connectome and not on the single-cell level. (6) Additionally, in case of polygenic disorders, the discrete phenotypic alterations might be very small, presented on a continuous spectrum. In contrast, iPSC/iNC studies usually recruit psychiatric patients with rare mutations of large effects. Selection of severe cases based on the polygenic score method could be a possible approach to overcome this limitation.

## 11. Take Home Messages

This review demonstrates the unprecedented possibilities offered by iPSC based* in vitro* disease modeling in psychiatry, a field of medicine awaiting major developments. It is striking how fragmented our understanding is about molecular disease pathways underlying SCZ, BPD, and ASD and how high the level of unmet needs is among patients suffering from these disorders. Incomplete therapeutic response, therapy-resistance, and cognitive deterioration are all major hurdles in the treatment of psychiatric patients.* In vitro* disease modeling will help us with diagnostics by demonstrating the heterogeneity within clinical disease groups in terms of molecular disease mechanisms. This question, whether similar clinical phenotypes share molecular foundations or are rather determined by a final common pathway, remains a central idea in psychiatry. Stem cells could also contribute to treatment by paving the way to personalized pharmacological treatment and drug screening as detailed in the review. The prospects of stem cell based disease modeling cannot be exactly foreseen; however, based on the past few years' developments we can envisage major breakthroughs in stem cell based psychiatry for the benefit of our patients.

## Figures and Tables

**Table 1:** Advancements and further challenges of the utility of induced pluripotent/neural cells in psychiatric research. The recommendations were conceived on a meeting of the National Institute of Mental Health and the Foundation for NIH in 2012 [10].

Recommendations in 2012	Advancements in the past 3 years	Challenges remain
Standardization of protocols		
(i) Optimizing reprogramming and differentiating methods (ii) Efficient generation and validation of specific neural cell types (iii) The importance of region and maturation state specific differentiation (iv) Poorly defined regional identity	(i) Safe, integration-free, nonviral induction (ii) Neurotransmitter and region specific protocols with efficacy >80% (iii) Multiple model based studies (iv) Combination of GWAS databases with iPSC/iNC observations	(i) Comparison of cells induced from different peripheral tissues (ii) Utility of induced glial cells in psychiatric research

Improving homogeneity		
(i) Detailed comparison of induced and source cells to reveal *de novo* genetic mutations (ii) Multiple parallel cell lines from one donor (iii) Epigenetic mapping during reprogramming and differentiation	(i) Vector integration-free, “safe” reprogramming methods (ii) Reassuring results on chromosomal mutations (iii) Average 3 cell lines/donor (iv) Experiments and reviews comparing the available protocols	(i) Concerns on *de novo* CNV mutations and the neuronal genome (ii) Contradictions regarding epigenetics (iii) Little is that known about endogenous production of astrocytes

Increasing statistical power		
(i) Increasing sample sizes (ii) Careful selection and grouping of subjects (iii) Detailed clinical and genetic characterization of subjects (iv) Overthought diseased-control pairing	(i) Studies with whole genome sequencing and whole transcriptome profiling (ii) Isogenic case-control comparison (new DNA editing techniques, twin studies)	(i) Increasing sample sizes (ii) Reconsideration of patient grouping (iii) Transparent, published case-control matching

Improve reproducibility, resource sharing, and collaboration		
(i) Establishing rigorous, transparent, and reproducible methods (ii) Detailed publication of protocols (iii) Rapid sharing of cell lines, technologies, and best practices (iv) Improving public-private partnership	(i) iPSC banks combined with gene banks (ii) Commercially available iPSCs, iCell neurons, and knock-out cell lines with isogenic controls (iii) Open access movements (iv) Results usually correlated with postmortem and animal model findings	(i) Guidelines for validation (ii) Criteria for cell characterization (markers, electrophysiological properties) (iii) Poor publication of donor's genotype, clinical features

Towards large-scale studies		
(i) Decreasing protocol diversity (ii) Validation assays for phenotypic comparison of derived cell lines	(i) Protocol diversity remains, but major steps towards large-scale production (ii) Commercially available cells provide enough experimental material for high throughput assays	Personalized medicine requires reprogramming and differentiation by every single patient, which is still remarkably time-consuming and money consuming

Careful patient selection, case-control matching		
(i) Subgrouping on the base of comprehensive genetic and clinical characterization (ii) Linking genotype with molecular and cellular pathophysiology	(i) Isogenic case-control pairs provided by DNA editing techniques, twin studies (ii) Pedigree-studies (iii) DSM-5 reconsidered subcategories	Endophenotype-based subgrouping?

**(a):** 

Author, publication date	Modeled disease(s)	Main findings	Cell line, differentiation protocol	Patient derived cell lines	Free full text
Ananiev et al., 2011 [11]	Rett syndrome	Neurons exhibit smaller nuclear size	Differentiated glutamatergic neurons	Y (3)	Y

Bavamian et al., 2015 [12]	BPD	miR-34a is associated with BPD and neurodevelopment	NPCs	Y (1)	N

Belinsky et al., 2014 [13]	Neurodevelopment	Electrophysiology and gene expression during neural maturation	Differentiated glutamatergic neurons	Y (1)	Y

Boissart et al., 2013 [14]	Psychopharmacology, ASD	Synchronous production of cortical neurons for high-throughput assays	Glutamatergic cortical neurons	Y (2)	Y

Brennand et al., 2011 [15]	SCZ	Diminished connectivity, cAMP and WNT signaling rescued by antipsychotic treatment	Panneuronal differentiation protocol, glutamatergic neurons	Y (4)	Y

Brennand et al., 2015 [16]	SCZ	Altered migration, mitochondrial damage, and increased oxidative stress	NPCs, panneuronal differentiation	Y (4)	Y

Brick et al., 2014 [17]	ASD	iPSC bank from ASD patients and controls	Differentiated glutamatergic neurons	Y (cell bank)	Y

Bundo et al., 2014 [18]	SCZ	LINE retroelements show more activity in SCZ derived cells	Differentiated glutamatergic neurons	Y (3)	Y

Chen et al., 2014 [19]	BPD	BPD iNCs exhibit Ca-signaling and neurodevelopment associated transcription alterations	Differentiated neurons (mixed glutamatergic-GABAergic populations)	Y (3)	Y

Chen et al., 2013 [20]	SCZ, BPD	Transcriptional effects of zinc finger protein 804A silencing	Differentiated glutamatergic neurons	N	Y

Cheung et al., 2011 [21]	Rett syndrome	Generation of MECP2 mutant iPSC/iNC lines and their isogenic pairs	Differentiated glutamatergic neurons	Y (1)	Y

Chiang et al., 2011 [22]	SCZ	Introduction of an integration-free method for reprogramming	iPSCs	Y (2)	Y

Corrales et al., 2012 [23]	SCZ	Copy number variations in iPSCs, iNCs	NPCs	Y^*∗*^	N

Dage et al., 2014 [24]	Psychopharmacology, ASD	Pharmacological and transcriptome characterization of iNCs	Forebrain neurons	N	N

DeRosa et al., 2012 [25]	ASD	iPSC and GABA neuron derivation from whole blood	GABAergic neurons	Y^*∗*^	Y

Doers et al., 2014 [26]	Fragile X syndrome	iNCs show neurite outgrowth deficit	Forebrain neurons	Y (3)	N

Germain et al., 2014 [27]	Neurodevelopmental disorders	Gene expression analysis of iPSCs from 15q11 variants	Differentiated glutamatergic neurons	Y (3)	Y

Griesi-Oliveira et al., 2014 [28]	ASD	TRPC6 gene is associated with ASD	Differentiated glutamatergic neurons	Y (1)	N

Hashimoto-Torii et al., 2014 [29]	SCZ	Heat shock transcription factor 1 mediated stress response abnormalities in a subpopulation of iNPCs	NPCs	Y (4)	Y

Hook et al., 2014 [30]	SCZ	Increased catecholaminerg neural activity in SCZ cell cultures	Panneuronal differentiation protocol	Y (4)	Y

Chung et al., 2014 [31]	Fragile X syndrome	Development of a high-content screening assay	NPCs	Y^*∗*^	N

Krey et al., 2013 [32]	Timothy syndrome	iNCs exhibit dendritic retraction deficit	Differentiated glutamatergic neurons	Y (2)	Y
Kumari et al., 2015 [33]	Fragile X syndrome	Development of a high-throughput screening assay	NPCs	Y (3)	N

Larimore et al., 2013 [34]	Rett syndrome	MECP2 regulates synaptic expression of dysbindin-BLOC1 pathway	Differentiated glutamatergic neurons	Y (2)	Y

Lin et al., 2012 [35]	SCZ	Allele specific expression profile	Differentiated glutamatergic neurons	Y (3)	Y

Lin et al., 2014 [36]	SCZ, ASD	Heat shock alters SCZ, ASD-related genes	3-dimensional neuronal aggregates	Y	Y

Liu et al., 2012 [37]	Fragile X syndrome	FMR1 mutation linked phenotype and signaling deficits	Differentiated neurons	Risk variant carrier	Y

Madison et al., 2015 [38]	BPD	Phenotypic alterations in BPD progenitors rescued by WNT inhibition	NPCs	Y (2) (pedigree-study)	N

Maekawa et al., 2015 [39]	SCZ, ASD	Hair follicle is a potential biomarker source	iPSC-derived neurospheres	Y	Y

Marchetto et al., 2010 [40]	Rett syndrome	Morphological and electrophysiological anomalies	Panneuronal differentiation protocol	Y	Y

Maschietto et al., 2015 [41]	SCZ	Altered gene expression profile during neurodevelopment	NPCs	Y (1)	Y

Niedringhaus et al., 2015 [42]	Fragile X syndrome	Mobile raft minicultures developed for high-throughput assays on neurons	Differentiated neurons in microraft cultures	Y (1)	Y

Paşca et al., 2011 [43]	Timothy syndrome	Disease-specific cellular phenotype and differentiation	Cortical glutamatergic neurons	Y (2)	Y

Paulsen et al., 2014 [44]	SCZ	Zinc and potassium imbalance reverted by valproate	NPCs	Y (2)	N

Pedrosa et al., 2011 [45]	SCZ	22q11.2 deletion delays differentiation	Glutamatergic neurons	Y (3)	N

Robicsek et al., 2013 [46]	SCZ	Impaired differentiation, maturation, and mitochondrial dysfunction	Dopaminergic neurons	Y (3)	N

Roussos et al., 2014 [47]	SCZ	CACNA1C variation disrupts gene regulation through chromosome loops	Differentiated glutamatergic neurons	N	Y

Shcheglovitov et al., 2013 [48]	22q13.3 deletion syndrome	SHANK3 and IGF1 correct excitatory synaptic transmission deficit	Differentiated glutamatergic neurons	Y (2)	N

Sheridan et al., 2011 [49]	Fragile X syndrome	Diminished neural differentiation	Differentiated neurons and glia	Y (3)	Y

Shi et al., 2014 [50]	Psychopharmacology	Dopamine 2 receptor is mediated by microRNA-9 and microRNA-326	Dopaminergic neurons	N	Y

Tian et al., 2014 [51]	Timothy syndrome	Altered Ca^2+^ signaling leads to transcriptional dysregulation	Differentiated glutamatergic neurons	Y (3)	Y

Topol et al., 2015 [52]	SCZ	Altered WNT signaling	Forebrain patterned NPCs	Y (4)	N

Wang et al., 2014 [53]	BPD	Cell adhesiveness is associated with lithium response	Immature iNs, lentiviral-based transdifferentiation	Y (12)	Y

Wen et al., 2014 [54]	SCZ, MDD	DISC1 mutation causes synaptic deficits and transcription dysregulation	Glutamatergic forebrain neurons	Y (2)	N

Williams et al., 2014 [55]	Rett syndrome	MECP2 mutant astrocyte influences negatively the morphology and function of cocultured neurons	Astrocytes	Y	Y
Yoon et al., 2014 [56]	SCZ	15q11.2 CNV results in neural stem cell deficit	Neural rosettes, NPCs	Y	Y

Yu et al., 2014 [57]	SCZ	Deficit in hippocampal granule neuron generation	Hippocampus dentate gyrus granule cells	Y (4)	Y

Zeng et al., 2013 [58]	Neurodevelopment	NRXN1 silencing impacts adhesion and differentiation related transcription	NPCs and differentiated neurons	N	Y

*∗* indicates data were not available.

ASD: autism spectrum disorders; BPD: bipolar disorder; iPSC: induced pluripotent stem cell; MDD: major depressive disorder; SCZ: schizophrenia; NPC: neural progenitor cell.

**(b):** 

Author(s) (31)	Year of publication	Disease(s)
Acab and Muotri	2015 [59]	ASD
Aigner et al.	2014 [60]	ASD
Ardhanareeswaran et al.	2015 [61]	ASD
Brennand and Gage	2012 [62]	Psychiatric disorders
Brennand et al.	2014 [63]	SCZ
Buxbaum and Sklar	2011 [64]	SCZ
Chailangkarn et al.	2012 [65]	Neurodevelopmental disorders
Cheung et al.	2012 [66]	Rett syndrome
Cundiff and Anderson	2011 [67]	Neuropsychiatric disorders
Duan	2015 [68]	SCZ
Freitas et al.	2014 [69]	ASD
Ho et al.	2015 [70]	Neuropsychiatric disorders
Cocks et al.	2014 [71]	ASD
Kim	2010 [72]	Psychiatric disorders
Kim et al.	2012 [73]	ASD
Kim et al.	2014 [74]	ASD
Ladran et al.	2013 [75]	Neuropsychiatric disorders
Liu and Scott	2014 [76]	ASD
Mackay-Sim et al.	2011 [77]	Neuropsychiatric disorders
Muotri	2015 [78]	ASD
O'Shea and McInnis	2015 [79]	BPD
Paşca et al.	2014 [80]	Neuropsychiatric disorders
Paulsen et al.	2012 [81]	SCZ
Paulsen et al.	2013 [82]	Neurodevelopmental disorders
Prilutsky et al.	2014 [83]	ASD
Qiang et al.	2014 [84]	Neuropsychiatric disorders
Schadt et al.	2014 [85]	Neuropsychiatric disorders
Tobe et al.	2013 [86]	Psychopharmacology
Tran et al.	2013 [87]	SCZ
Vaccarino et al.	2011 [88]	Neuropsychiatric disorders
Viswanath et al.	2015 [89]	BPD
Walsh and Hochedlinger	2010 [90]	Rett syndrome
Wright et al.	2014 [91]	SCZ

ASD: autism spectrum disorders; BPD: bipolar disorder; SCZ: schizophrenia.
